# “It pains me because as a woman you have to breastfeed your baby”: decision-making about infant feeding among African women living with HIV in the UK

**DOI:** 10.1136/sextrans-2015-052224

**Published:** 2016-01-12

**Authors:** Shema Tariq, Jonathan Elford, Pat Tookey, Jane Anderson, Annemiek de Ruiter, Rebecca O'Connell, Alexandra Pillen

**Affiliations:** 1 School of Health Sciences, City University London, London, UK; 2 Department of Anthropology, University College London, London, UK; 3 MRC Centre of Epidemiology for Child Health, UCL Institute of Child Health, London, UK; 4 Centre for the Study of Sexual Health and HIV, Homerton University Hospital NHS Foundation Trust, London, UK; 5 Harrison Wing, Guy's and St Thomas’ NHS Foundation Trust, London, UK; 6 Greenway Centre, Newham University Hospital, London, UK

**Keywords:** AIDS, CULTURAL DIFFERENCE, HIV WOMEN, PREGNANCY, QUALITATIVE RESEARCH

## Abstract

**Objectives:**

UK guidance advises HIV-positive women to abstain from breast feeding. Although this eliminates the risk of postnatal vertical transmission of HIV, the impact of replacement feeding on mothers is often overlooked. This qualitative study examines, for the first time in the UK, decision-making about infant feeding among African women living with HIV.

**Methods:**

Between 2010 and 2011, we conducted semistructured interviews with 23 HIV-positive African women who were pregnant or had recently given birth. We recruited participants from three HIV antenatal clinics in London.

**Results:**

Women highlighted the cultural importance of breast feeding in African communities and the social pressure to breast feed, also describing fears that replacement feeding would signify their HIV status. Participants had significant concerns about physical and psychological effects of replacement feeding on their child and felt their identity as good mothers was compromised by not breast feeding. However, almost all chose to refrain from breast feeding, driven by the desire to minimise vertical transmission risk. Participants’ resilience was strengthened by financial assistance with replacement feeding, examples of healthy formula-fed children and support from partners, family, peers and professionals.

**Conclusions:**

The decision to avoid breast feeding came at considerable emotional cost to participants. Professionals should be aware of the difficulties encountered by HIV-positive women in refraining from breast feeding, especially those from migrant African communities where breast feeding is culturally normative. Appropriate financial and emotional support increases women's capacity to adhere to their infant-feeding decisions and may reduce the emotional impact.

## Introduction

This risk of transmitting HIV through breast feeding is approximately 16% in the absence of antiretroviral therapy (ART).^1^ Exclusive replacement feeding (feeding an infant milk other than breast milk, such as formula) eliminates the risk of vertical transmission through breast feeding. However, in low-income settings it presents significant operational challenges and is associated with increased infant mortality secondary to diarrhoeal illness and malnutrition, when compared with exclusive breast feeding in HIV-positive mothers.^2^ ^3^ WHO guidelines therefore recommend that the majority of mothers living with HIV in low-income settings exclusively breast feed with the provision of ART to mother or child reducing the transmission risk to between 0% and 6%.^4^ ^5^

In high-income settings such as the UK, widespread access to clean water and formula means that the risk of vertical transmission of HIV associated with breast feeding outweighs that of infant malnutrition and diarrhoeal illness. As a result, UK guidance recommends exclusive replacement feeding for all women diagnosed with HIV.^6^ ^7^ Where a mother has an undetectable viral load on ART and chooses to breast feed, exclusive breast feeding, careful monitoring of both mother and child and cessation of breast feeding by 6 months are advised.^7^

Between 1200 and 1400 pregnancies are reported in the UK each year among women with diagnosed HIV infection (http://www.ucl.ac.uk/silva/nshpc/slides, accessed 06 May 2015), with one of the lowest vertical transmission rates globally at <0.5%.^8^ Little is known about factors that shape infant-feeding decisions in women living with HIV in the UK, the vast majority of whom have migrated from sub-Saharan Africa.^9^ Numerous studies conducted in sub-Saharan Africa have highlighted the structural and sociocultural constraints experienced by HIV-positive women when making infant-feeding decisions, and it is widely acknowledged that in many of these settings these decisions are highly conflicted.^10^ Previous case reports and small studies suggest that the avoidance of breast feeding is also challenging for women in the UK and the US, especially those from migrant African communities.^11–13^

In this paper, we present the first qualitative study in the UK to explore, in detail, decision-making about infant feeding among mothers living with HIV, with specific reference to migrant African women.

## Methods

We present an analysis of qualitative data obtained from semistructured interviews drawn from a larger mixed-methods observational study designed to explore HIV-positive African women's engagement with HIV care during and after pregnancy. The full methods of the larger study are described elsewhere.^9^

Twenty-three pregnant women were recruited from three specialist NHS HIV antenatal clinics in London. These three sites were among the five hospitals reporting the largest numbers of pregnancies in HIV-positive women between 2000 and 2010 (data extracted from the National Study of HIV in Pregnancy and Childhood, available from authors on request), each site looking after approximately 40–50 pregnant women living with HIV annually. Since the first published UK guidelines on management of HIV in pregnancy in 2001, HIV-positive mothers have been advised to completely abstain from breast feeding. Interviews were conducted between October 2010 and October 2011. During this time, the British HIV Association published a position statement reinforcing the advice to abstain from breast feeding, but for the first time explicitly stating that breast feeding against medical advice in the context of effective ART and maternal virological suppression was not grounds for automatic referral to child protection services.^7^

Healthcare professionals working at each study site identified and approached women attending for HIV antenatal care who were eligible for the study. Women were eligible if they were of black African ethnicity, were born in sub-Saharan Africa, were diagnosed with HIV and were pregnant (at any gestation). Of the 23 women who agreed to take part in the study, one chose to defer her interview until after delivery due to poor health. Sampling was purposive in order to recruit women from a range of African regions, with a range of migration histories and with different durations of HIV diagnosis (see table 1 for sociodemographic characteristics of participants). Recruitment continued until we reached data saturation. Written informed consent was obtained from all participants.

**Table 1:** Interview participant characteristics

Characteristic	Number of participants (n=23)
African region of birth	
East Africa	10
West Africa	10
Southern Africa	0
Middle Africa	3
Highest level of education completed*	
Primary	1
Secondary	9
Higher	13
Employed	
No	9
Yes	14
Marital status	
Married/cohabiting	8
In a non-cohabiting relationship	8
Single	7
Duration of residence in UK (years)	
<1	1
1–4	4
5–10	12
>10	6
Immigration status†	
Insecure	9
Secure	14
Diagnosis of HIV	
Prior to current pregnancy	20
During current pregnancy	3
Children prior to current pregnancy	
Yes	20
No	3

*Secondary education was defined as up to secondary school. Higher education was defined as college or university education (including higher professional qualifications).

†Secure immigration status was defined as being a UK citizen, a recognised refugee or having exceptional or indefinite leave to remain. Anyone not in these categories was defined as having insecure immigration status.

**Figure 1:**
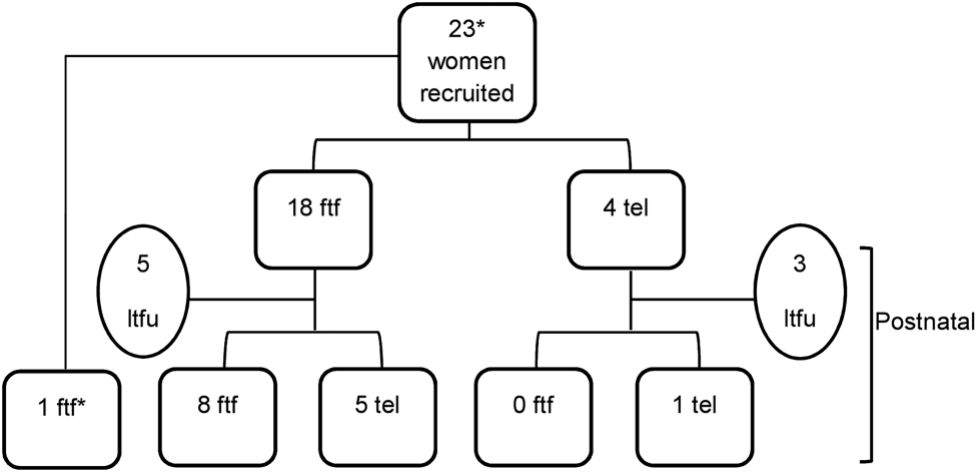
Recruitment and follow-up of interview participants. *One woman was recruited and interviewed postnatally face to face. ftf, face-to-face interview; tel, telephone interview; ltfu, lost to follow-up.

Initial face-to-face interviews (n=19) were conducted within clinics by the first author with an interpreter present if required (n=1). Topics included experiences of pregnancy, attitudes to medical interventions, psychosocial support, experiences of healthcare during pregnancy and experiences of stigma and discrimination. A minority of initial interviews (n=4) were conducted by telephone due to participant preference (figure 1). The 22 women who had been interviewed during pregnancy were invited to participate in a second interview within 12 months of giving birth, of whom 14 consented to be interviewed a second time (figure 1).

Interviews were audio-recorded unless participants expressed objections. In these rare cases (n=2), extensive contemporaneous written notes were taken. All interviews were transcribed verbatim and analysed in NVivo V.9.0 (QSR International, 2010). The first author undertook a thematic analysis of interview data, using the constant comparative method usually associated with grounded theory.^14^ This is an inductive process where each transcript is read several times and sections of the text coded within the database. We began with initial open coding, a process where codes are identified from data without restriction. Coded text was then compared and linked across all the interviews leading to the development of broader thematic categories. For the purposes of this analysis, we reduced the data by extracting text that referred to infant feeding.

The codebook and explanatory framework were developed by the first author in discussion with AP. Emergent findings were also presented to a forum of women living with HIV at one of the collaborating sites in order to ascertain the validity of data interpretation.

## Theoretical framework

The notion of risk as socially constructed, as outlined by Douglas and Wildavsky,^15^ is a useful framework for understanding decision-making about infant feeding in the context of HIV. This approach recognises that the perception of risk is dependent upon context and that decisions about risk are socially embedded. This is in contrast to the *technical approach* to risk, the dominant paradigm in public health.^16^ The technical approach assumes that decisions about risk are made by an individual who is free to make choices, that these are rational choices underpinned by a value-free cost-versus-benefit analysis and that those who do not subscribe to the consensus about dangers are irrational or poorly informed.^15^ ^17^

The social construction of risk has informed previous HIV prevention research, generating important insights into ‘high risk’ behaviours such as barebacking^18^ and sexual and injecting risk-taking among people who use drugs.^19^ In this paper, we draw upon this theoretical framework to explore the social meanings of both breast feeding and replacement feeding for women living with HIV, how participants’ decisions about infant feeding are located within a social domain and the various factors that constrain their capacity to make choices.

## Results

Of the 15 women interviewed postnatally, only one described breast feeding her infant. Of the remaining eight women who were *not* interviewed postnatally, all stated their intention to avoid breast feeding completely. Participants were knowledgeable about the risks of breast feeding and emphasised the paramount importance of minimising the risk of HIV transmission to their child. However, the decision to abstain from breast feeding was often fraught and characterised by feelings of guilt, sorrow and fear. Of the 23 women interviewed, only 4 women appeared not to be conflicted about their choice to formula-feed their infant. In the following sections, we refer to illustrative quotes (box 1) from interviews by number.

Box 1 **Illustrative quotes**

1. Difficulties in avoiding breast feeding
  a. Emotional impact of avoiding breast feeding
    i. Emotional cost: *Even up to this very day I feel bad I've not been able to give him the best, you know, natural way of feeding. That kind of hits me in a way.* (306, 36, married, PNI)
    ii. Reminder of HIV status: *At times you want to forget this thing, HIV. If you keep explaining you have to tell lies within yourself. It's a kind of emotional trauma.* (202, 38, relationship, ANI)
    iii. Fears about infant health: *I wanted to [breastfeed], because from what I read in all the books, breast milk was the best, so I wanted to give my baby.* (209, 33, married, PNI)
    iv. Concerns about infant bonding: *When a baby doesn't breastfeed, it doesn't listen to you. The baby that breastfeeds, you know, they have more love than the baby who doesn't.* (200, 30, relationship, ANI)
  b. Practical difficulties
    i. Preparation of feed: *It's a lot of work. All the time keep sterilising, washing, boiling water, it's not easy. During the nights I'll get up stressed, because I have to get up and boil water all those times if he cries, or if he needs to drink.* (204, 29, married, PNI)
    ii. Lack of financial resources: *The formula as well is very expensive and you're thinking, ‘Oh my God, if I was breastfeeding I wouldn't be spending so much money on just buying milk all the time.’ At some point [in a previous pregnancy] we could not afford it […] it was so expensive.* (304, 28, married, ANI)
  c. Social barriers
    i. Cultural value of breast feeding: *Sometimes I feel it's so hard … that's what really makes me feel sad when I look at my baby, as a black woman, because in our culture in Africa you're supposed to breastfeed for two years.* (207, 27, single, ANI)
    ii. Maternal identity: *I just accept it but in my heart it pains me because as a woman you have to breastfeed your baby.* (301, 42, married, ANI)
    iii. Social pressure: *My church members they ask questions too much […] they want to know everything. They have a mothers’ room where you can go with your baby. When everybody was there and feeding their baby [they ask] ‘why you don't want to breast feed?’* (103, 39, married, ANI)
    iv. Disclosure of HV status: *You always have to explain that this baby is taking medication and then that you're not breastfeeding. So everything adds up and you know it's [suspicions about her HIV-status] just going to be out there.* (304, 28, married, ANI)
2. Breast feeding against advice
  *My doctor was telling me everything was OK with my blood; that it's undetectable. So I thought maybe I should just try. I know it's not possible for the baby to get the virus except during birth*.
  (303, 25, relationship, PNI)
3. Fostering resilience
  a. Support from others: *My husband tells [others] that ‘not every women always like breastfeeds, I don't know if she's shy to show her breasts outside’.* (204, 29, married, PNI)
  b. Previous experience of not breast feeding: *My first daughter wasn't breastfed and she's okay so this one now is not going to be breastfed.* (208, 42, single, ANI)
  c. Financial assistance:
    *Respondent: I was given the voucher to buy some milk and I will get a voucher every month as well*.
    Interviewer: Does that help?
    *Respondent: Yes, it does. [The clinic] is very kind.* (208, 42, single, ANI)

**(participant ID, age, relationship status, antenatal (ANI) or postnatal (PNI) interview)**

### Difficulties in avoiding breast feeding

#### Emotional impact

In abstaining from breast feeding, participants felt an acute sense of personal loss, often crying when discussing their decision and describing themselves as ‘sad’, ‘unhappy’ and ‘devastated’. It was clear that avoiding breast feeding came at a significant emotional cost (1.a.i). Furthermore, the decision not to breast feed was not an isolated event; rather it was a process that continued throughout their child's infancy. Women often found themselves revisiting their decision either on feeding their baby or when questioned by others as to why they were not breast feeding. This served as a painful reminder of their HIV-positive status (1.a.ii). Many participants were concerned that abstaining from breast feeding would lead to poor infant health and affect their ability to bond with their child (1.a.iii, 1.a.iv).

#### Practical difficulties

Women highlighted the work involved in replacement feeding in terms of preparing equipment and formula (1.b.i). For those who were sole caregivers, the practical demands of replacement feeding without the support of a partner were especially great. Furthermore on migrating to the UK, many had left behind support networks that they could ordinarily draw support from. An even greater challenge was the significant cost of formula (1.b.ii). For women who did not qualify for state-funded financial assistance, buying formula often put a strain on household finances. Those with insecure immigration status and no recourse to public funds found themselves in an even more precarious situation. They were often financially dependent on friends, family and partners, who were sometimes unaware of their HIV status and were unwilling to provide financial assistance.

#### Social barriers

Participants felt a strong cultural imperative to breast feed, especially as African women coming from cultures where breast feeding is highly valued (1.c.i). For many, breast feeding was intrinsically tied to being both a good woman and a good mother (1.c.ii). Infant-feeding decisions were deeply embedded within women's social networks. They described being scrutinised and their feeding decisions commented upon (1.c.iii). Furthermore, avoiding breast feeding also raised suspicions about their HIV-positive status, which women were reluctant to disclose due to widespread HIV-related stigma (1.c.iv). With public breast feeding common in many participants’ communities, the bottle-fed infant therefore functioned as a visible surrogate marker for an HIV-positive status. Finally, as part of the African Diaspora in the UK, our participants were particularly aware of the differences in international public health policy on HIV and infant feeding. Through the strong links they maintained with their home countries, many knew of HIV-positive women who had been advised to breast feed or had themselves received conflicting advice from medical staff in Africa and in the UK. This discrepancy was a source of confusion for some participants, prompting them to question the validity of the advice they were receiving.

### Breast feeding against advice

Only one participant in this study disclosed that she had breast fed her child. Her social situation was precarious in that she was a single mother of two with no leave to remain in the UK and therefore no recourse to public funds. She lived with her family, to whom she had not disclosed her HIV status, and was financially dependent on them. On the birth of her second child, she had decided to supplement formula with breast feeding, satisfied that her HIV was well controlled and believing that there was no risk of HIV transmission (2).

Her decision to breast feed was partly underpinned by her own understanding of the risk of vertical transmission. However, her decision was largely socially embedded, shaped by financial dependency on her family and the need to keep her HIV-status hidden. She had not discussed her decision to breast feed with her medical team because “they said I shouldn't do it.” However, the absence of an open discussion with clinicians about her feeding intentions had resulted in the participant practicing mixed feeding, rather than breast feeding with appropriate monitoring which would have been the safer option in this complex situation.

### Fostering resilience

Despite the challenges outlined above, the overwhelming majority of participants adhered to their decision to abstain from breast feeding. Women who had disclosed their HIV status to partners or family members, and had their support, found it easier to adhere to their decision as they often received practical and financial assistance, as well as validation of their decision in front of others (3.a.). Those who had not breast fed previous children and seen them thrive also displayed more confidence in subsequent decisions to avoid breast feeding (3.b.). Finally, the provision of free milk vouchers and equipment through clinics was a way of supporting women, many of whom were socially vulnerable, to exclusively feed their infants formula (3.c.).

## Discussion

Since 2001, UK guidance has continued to advise mothers living with HIV to completely abstain from breast feeding in line with guidelines in other high-income settings such as the US and France.^20^ ^21^ This is the first qualitative study in the UK to explore, in detail, infant-feeding decisions made by women living with HIV, and one of the first studies to be conducted internationally in a high-income setting. Informed by theories on the social construction of risk, our analysis highlights how our participants’ infant-feeding decisions were socially embedded. Although their decisions were driven by the desire to secure the health of their babies, they were subject to a range of competing concerns including cultural expectations of motherhood, relationships within their social networks and economic and social vulnerability.

The complexity of infant-feeding decisions in the context of HIV is widely recognised throughout sub-Saharan Africa. Studies have revealed how decision-making about infant feeding in many African countries extend beyond the mother–child dyad, with spouses, extended family and the wider local community all contributing to decisions.^22–24^ In these settings, where breast feeding is the only culturally acceptable form of infant feeding, abstaining from breast feeding arouses suspicion and may function as a surrogate marker of a mother's HIV-positive status.^25–28^

Our work demonstrates how HIV-positive women's decisions to abstain from breast feeding in high-income settings can be similarly complex, echoing findings from previous case reports and small studies conducted in the UK and the North America.^11–13^ Although the considerable infrastructural barriers to replacement feeding faced by women in low-income settings may not be encountered in countries such as the UK, our participants were still subjected to economic constraints and sociocultural pressures to breast feed. Furthermore, our study highlights how strategies to prevent vertical transmission of HIV dominate pregnancy for women living with HIV and also continue into the postnatal period, often at significant emotional cost.

Where our study differs from those conducted in low-income settings is in its focus on migrant women. Our participants’ location within the African Diaspora gave them an acute awareness of international variations in public health policy on HIV and infant feeding, sometimes resulting in confusion. Furthermore, the poverty and lack of citizenship rights that often accompany migration forced some of our participants into economic dependency, presenting a further challenge to their ability to abstain from breast feeding.

Our findings should be interpreted with caution. This study focused on African migrant women and may not reflect the experiences of women from other cultural groups. Participants were recruited in London and their experience may differ from those in other areas where there is likely to be less access to specialist clinical and support services. Furthermore, in recruiting participants from NHS clinics we accept that our findings are unlikely to capture the experiences of women who are poorly engaged with clinical services.

However, this is one of very few qualitative studies to date of infant feeding among women living with HIV in a high-income setting, allowing us to suggest ways in which professionals can strengthen HIV-positive mothers’ resilience.

The majority of HIV-positive women in high-income settings are likely to abstain from breast feeding in order to minimise the risk of vertical transmission. Healthcare professionals should continue to be aware of the difficulties encountered by women in adhering to guidance to abstain from breast feeding, and strive to create an environment where women feel able to express their concerns openly. Furthermore, staff should be prepared to explicitly and clearly address the discrepancies between national and international guidance on HIV and breast feeding in order to minimise confusion. Healthcare professionals, partners, family and peer ‘mentor-mothers’^29^ have an important supportive role. This includes addressing women's concerns about the impact of exclusive replacement feeding on infant health and providing advice on bonding, such as maintaining skin-to-skin contact. Lastly, the provision of free formula and sterilising equipment should be guaranteed across all UK clinics in order to allow HIV-positive women, many of whom are socioeconomically marginalised, to feed their babies safely without having to disclose their HIV status to those they are economically dependent upon.

Antenatal teams should continue to provide opportunities for open and non-judgemental conversations about breastfeeding intentions, and be prepared to support the minority of women who choose to breast feed to do so safely, in line with current UK guidance on infant feeding and HIV.^7^ As further data emerge to support breast feeding in the context of maternal virological suppression, increasing numbers of women living with HIV may choose to breast feed. Finally, teams should be aware that exclusive breast feeding could also be challenging in communities where mixed feeding may be the cultural norm,^23^ ^30^ and that women may need specific counselling and education around this.

In conclusion, this study highlights that adherence to national guidance to abstain from breast feeding can be fraught and complex for HIV-positive African migrant women living in the UK. The onus is on healthcare professionals to understand the difficulties HIV-positive mothers may encounter and to provide material and psychosocial support in order to promote maternal well-being and prevent disengagement from HIV care.

**Key messages**

- In high-income settings such as the UK, mothers living with HIV are advised to abstain from breast feeding to prevent vertical transmission of HIV to their infants.
- This is the first qualitative study in a high-income setting to explore the impact of mode of infant feeding on women living with HIV.
- In this study, which focuses on migrant African women, all but one woman chose to refrain from breast feeding; however, this came at considerable emotional cost.
- Financial assistance with replacement feeding and appropriate support from partners, family, peers and healthcare providers strengthens women's resilience in maintaining their decision not to breast feed.
